# Chromium Stress Mitigation by Polyamine-Brassinosteroid Application Involves Phytohormonal and Physiological Strategies in *Raphanus sativus* L.

**DOI:** 10.1371/journal.pone.0033210

**Published:** 2012-03-29

**Authors:** Sikander Pal Choudhary, Mukesh Kanwar, Renu Bhardwaj, Jing-Quan Yu, Lam-Son Phan Tran

**Affiliations:** 1 Department of Horticulture, Zhejiang University, Hangzhou, Zhejiang, China; 2 Department of Botany, University of Jammu, Jammu, India; 3 Department of Botanical and Environmental Sciences, Guru Nanak Dev University, Amritsar, Punjab, India; 4 Signaling Pathway Research Unit, RIKEN Plant Science Center, Yokohama, Kanagawa, Japan; National Taiwan University, Taiwan

## Abstract

Brassinosteroids (BRs) and polyamines (PAs) are well-established growth regulators playing key roles in stress management among plants. In the present study, we evaluated the effects of epibrassinolide (EBL, an active BR) and spermidine (Spd, an active PA) on the tolerance of radish to oxidative stress induced by Cr (VI) metal. Our investigation aimed to study the impacts of EBL (10^−9^ M) and/or Spd (1 mM) on the biochemical and physiological responses of radish (*Raphanus sativus* L.) under Cr-stress. Applications of EBL and/or Spd were found to improve growth of Cr-stressed seedlings in terms of root length, shoot length and fresh weight. Our data also indicated that applications of EBL and Spd have significant impacts, particularly when applied together, on the endogenous titers of PAs, free and bound forms of IAA and ABA in seedlings treated with Cr-stress. Additionally, co-applications of EBL and Spd modulated more remarkably the titers of antioxidants (glutathione, ascorbic acid, proline, glycine betaine and total phenol) and activities of antioxidant enzymes (guaicol peroxidase, catalase, superoxide dismutase and glutathione reductase) in Cr-stressed plants than their individual applications. Attenuation of Cr-stress by EBL and/or Spd (more efficient with EBL and Spd combination) was also supported by enhanced values of stress indices, such as phytochelatins, photosynthetic pigments and total soluble sugars, and reduction in malondialdehyde and H_2_O_2_ levels in Cr-treated seedlings. Diminution of ROS production and enhanced ROS scavenging capacities were also noted for EBL and/or Spd under Cr-stress. However, no significant reduction in Cr uptake was observed for co-application of EBL and Spd when compared to their individual treatments in Cr-stressed seedlings. Taken together, our results demonstrate that co-applications of EBL and Spd are more effective than their independent treatments in lowering the Cr-induced oxidative stress in radish, leading to improved growth of radish seedlings under Cr-stress.

## Introduction

Chromium (Cr) contamination of arable land and water bodies has severely raised the menace of this toxic element in the last few decades. Cr (III or VI) is not required by plants for their normal plant metabolic activities. On the contrary, excess of Cr (III or VI) in agricultural soils causes oxidative stress to many crops. Easy penetration of Cr (III or VI) in human food chain has made them a potent carcinogen. Anthropogenic release of Cr (III or VI) from leather, electroplating, chromic acid production and refractory steel industries constitutes main sources of Cr pollution [1]–[4]. Cr-induced oxidative stress in plants is reflected by reduced CO_2_ fixation and photosynthesis, inhibition of electron transport, inactivation of calvin cycle enzymes and chloroplast disorganization [1], [5]. Reactive oxygen species (ROS), like H_2_O_2_, OH^•^ and O_2_^−^ generated under Cr-stress, are highly reactive and cause oxidative damages to DNA, RNA, proteins and pigments [3], [6].

Plants combat oxidative stress with a unique organization of hydrophilic and lipophilic compounds (tocopherol, carotene, phenols) along with an array of antioxidant enzymes, such as guaiacol peroxidase (GPOX), catalase (CAT), glutathione reductase (GR) and superoxide dismutase (SOD) [7]–[9]. Metal detoxification in plant body is also achieved through active participation of phytochelatins (PC) and metalothionenins (MT) [10]. Recent research advances have shown the promising effects of plant growth regulators (PGRs) like auxins, abscisic acid (ABA), cytokinins, gibberellins, brassinosteroids (BRs) and polyamines (PAs) in abiotic stress mitigation [11]–[18]. Radish (*Raphanus sativus* L.) is a favorite root vegetable widely consumed for its high antioxidant, antidiabetic, antihepatotoxic and cholertic properties [19]. Economic loss to radish growers under Cr excess has been evidenced due to reduction in yield and dietetic values [1].

Among PGRs, BRs form a group of steroidal lactones with a wide array of roles in physiological activities, such as stem elongation, xylem differentiation, leaf bending, epinasty, pollen tube growth, fruit development, ethylene biosynthesis, photosynthesis and proton-pump activation [20]. Furthermore, ability of BRs to boost antioxidant system of plants is extensively used to confer resistance in plants against a variety of stresses, such as thermal, drought, heavy metal, pesticides, salinity, fungal and viral stresses [20], [21]. More recently, interactions of BRs with other PGRs, such as auxins, ABA, gibberellins and ethylene, have also been found to play a major role in plant stress alleviation [11].

PAs are small aliphatic nitrogenous compounds with ubiquitous distribution [12]. Implication of PAs in amelioration of various abiotic and biotic stresses has made them an essential component of plant defense mechanism [12]. Enhanced expression of spermidine (Spd) synthase (SPDS) and Spd titers has been associated with improved heavy metal and salinity tolerance in three transgenic European pears [22].

Latest developments in plant hormonal interactions have shown possible involvement of PGRs in abiotic stress management [23], [24]. However, scant information is available on the co-application effects of BRs and PAs on the heavy metal stress mitigation. Therefore, present investigation will aim to determine the impacts of BR and PA co-application on metal stress management in radish. We evaluated the effects of 24-epibrassinolide (EBL), an active BR, and spermidine (Spd), an active PA, on the contents of endogenous PAs, auxins and ABA as well as on the antioxidant systems, stress markers and growth parameters in seedlings of radish grown under Cr (VI) stress. Our results indicate that co-applications of EBL and Spd have remarkable influence on Cr (VI) stress mitigation than their individual applications.

## Materials and Methods

Certified and disease-free seeds of *R. sativus* L. cv. ‘Pusa chetki’ used in the present investigation were procured from Punjab Agriculture University, Ludhiana, India. After surface sterilization, seeds were grown in autoclaved Petri dishes lined with Whatman No. 1 filter paper at 25°C with a 16 h photoperiod under fluorescent white light (175 µmol/m^2^/s) in a controlled environmental growth chamber with 75% relative humidity.

### EBL, Spd and Cr (VI) treatments

Seeds of *R. sativus* were treated with either EBL (10^−9^ M) or Spd (1 mM) alone or in their respective combinations with 1.2 mM Cr (VI) (K_2_CrO_4_) solution. Seedlings (hypocotyls 3.5–4 cm long) were harvested on the 7^th^ day for determination of growth parameters or weighed and stored at −20°C until further analyses. Seedlings were used to study the possible interactive effects of EBL and Spd co-applications or individual treatments on endogenous titers of PAs, auxin (indole-3-acetic acid, IAA), ABA and antioxidant defense system and stress indicators. Cr (VI) concentration used in the present investigation was the IC_50_ value of Cr (VI) determined for *R. sativus* seedlings in germination and growth tests [6].

### Morphological parameters

Seven-day-old treated or untreated seedlings were harvested, and their shoot length (SL), root length (RL) and fresh weight (FW) were determined.

### Estimation of endogenous IAA, ABA and PA profiles

Endogenous titers of IAA and ABA were determined by the methods previously reported in [25] and [26], respectively. The estimation of PAs was carried out as previously described [27].

### Estimation of protein content and antioxidant enzyme activities

#### Preparation of plant extracts

Approximately 2 g FW of 7-d-old seedlings were homogenized with pre-chilled pestle and mortar in 6 ml of 0.1 mM potassium phosphate buffer (pH 7.0) under ice-cold conditions. The protein content was determined by Lowry's method [28].

#### Endogenous titers of antioxidants

Standard methods were followed for the estimation of endogenous levels of antioxidants, such as glutathione (GSH) [29], proline (PL) [30], ascorbic acid (ASA) [31], glycine betaine (GB) [32] and total phenols (TP) [33].

#### Estimation of antioxidant enzyme activities

Activities of antioxidant enzymes were determined by standard methods as previously described in [34] for GPOX (EC 1.11.1.7), [35] for CAT (EC 1.11.1.6), [36] for SOD (EC 1.15.1.1) and [37] for GR (EC 1.6.4.2).

### Stress indices

Stress markers used to evaluate the intensity of Cr-stress, including phytochelatins (PC) [38], photosynthetic pigments (PP) [39], malondialdehyde (MDA) [40], total soluble sugars (TSS) [41] and H_2_O_2_ content [42], were determined as previously described.

### Analysis of photosystem (PS) II quantum yield

PS II quantum yield was determined with an imaging pulse amplitude modulated fluorometer (IMAG-MAXI; HeinzWalz) and calculated by the method described in [43].

### Histochemical staining of O_2_^−^ and H_2_O_2_

Localization of O_2_^−^ and H_2_O_2_ was performed using the nitroblue tetrazolium (NBT) and diaminobenzidine (DAB) staining methods as described in [44], [45].

### Plasma membrane isolation and NADPH oxidase activity

Plasma membrane (PM) was isolated from seedlings (2 g FW) with two-phase aqueous polymer partition system. The NADPH oxidase activity was determined as previously described [46].

### Determination of Cr content

Cr content in seedlings (2 g dry weight, DW) was determined by using 10% *v/v* HNO_3_ acid digestion procedure as described in [47] and expressed as µg g^−1^ DW.

### Estimation of ion leakage

To assess the membrane damage caused by Cr-stress and loss of membrane permeability, ion leakage assay was performed as described in [48].

### Free radical scavenging activities

Standard assays for 1,1-diphenylpicrylhydrazyl (DPPH) [49], deoxyribose [50], [51] and Fe^2+^ ion reducing antioxidant power (FRAP) [52] were performed as previously described.

### RNA extraction and gene expression analysis

Total RNA was extracted from whole seedlings using TRIzol method according to supplier's instructions (Invitrogen). About 1 µg of total RNA was reverse-transcribed. The reaction mixture consisted of 0.5 µg of oligo (dT)_12–18_ (Invitrogen) and 200 units of Superscript II (Invitrogen) was used according to the manufacturer's supplied protocol. Quantitative real-time PCR (qRT-PCR) was performed using the iCycler iQ real-time PCR detection system (Bio-Rad) and the SYBR Green PCR Master Mix (Applied Biosystems) with the following thermal profile: denaturation at 94°C for 3 min, followed by 40 cycles at 94°C for 30 s, 58°C for 30 s and 72°C for 30 s. A dissociation curve was generated at the end of each PCR cycle to verify that a single product was amplified using software provided with the iCycler iQ real-time PCR detection system. The *26S rRNA* (AY366932.1) was used as a reference gene in the expression analysis. All experiments were repeated three times for cDNAs prepared from two samples of radish tissues. The quantification of mRNA levels was based on the method of Livak and Schmittgen [53]. The threshold cycle (Ct) value of *26S rRNA* was subtracted from that of the gene of interest to obtain a ΔCt value. The Ct value of untreated control sample was subsequently subtracted from the ΔCt value to obtain a ΔΔCt value. The fold changes in expression level relative to the untreated control were expressed as 2^−ΔΔCt^. Specific primer pairs were designed for *26S rRNA* (F: 5′-GCCCTCCTACTCATCGA-3′ and R: 5′-CACCTGCCGAATCAACT-3′), *RsADC* (FD 955649.1) (F: 5′-GGCTGTTGTGGCGTCTGTTA-3′, R: 5′-GCTTCCTCTGAGTACCCTTC-3′) encoding arginine decarboxylase and *RsSPDS* (FD583934.1) (F: 5′-CAACTTCCCGCAATACACCTC-3′ and R: 5′-CTCTATCCCTAACCCAAAG-3′) encoding spermidine synthase using the respective ESTs obtained from www.plantgdb.org. Putative *RsADC* and *RsSPDS* from *R. sativus* were identified by using the *AtADC* and *AtSPDS* of *Arabidopsis thaliana* for homology search against the EST databank of *R. sativus* available at www.plantgdb.org.

### Statistical analysis

All the experiments were performed in triplicates. The data shown are the means of three replicate experiments along with standard error (n = 3). One-way analysis of variance (ANOVA) was carried out and data were presented at p<0.05. All the statistical calculations were performed using Sigma Stat 3.5.

## Results

### EBL and Spd improve seedling growth under Cr-stress

Strong inhibition of seedling growth under Cr-stress was observed when compared with untreated control. Approximately 2.33-fold decrease in RL and 2.79-fold decrease in SL were observed for Cr-stressed seedlings over untreated control (CN) (Table 1). Application of EBL with Cr-solution was able to improve RL (1.64-fold) and SL (1.95-fold) in comparison with Cr-stress alone. Enrichment of Cr-solution with only Spd was also able to increase RL and SL by 1.34- and 1.91-fold, respectively, when compared with Cr-stressed seedlings (Table 1). In contrast, EBL+Spd combination was only effective in improving the SL by 2.2-fold over Cr-stress alone, while no significant change in RL was recorded. Significant decrease in FW (2.17-fold) of Cr-stressed seedlings was observed over untreated control. Cr-stressed seedlings fed with only EBL could improve their FW by 1.88 folds when compared with Cr- treatment alone. Enrichment of Cr-solution with only Spd also showed an increase in FW by 1.52-fold in comparison with Cr-stressed seedlings. Combined application of EBL and Spd with Cr-solution was more effective than individual applications in increasing FW as shown by a 2.12-fold increase over Cr-stress alone (Table 1). Collectively, our results demonstrate that EBL and Spd, either alone or together, can improve seedling growth under Cr-stress. However, co-application of EBL and Spd could improve growth parameters under Cr-stress more effectively than individual applications of EBL or Spd.

**Table 1:** Effect of EBL and/or Spd on morphological parameters of radish seedlings under Cr-stress.

Treatment	Root length (cm)	Shoot length (cm)	Fresh weight (g)
Control	6.90±0.192^a^	4.13±0.186^a^	0.270±0.005^a^
Cr	2.96±0.071^b^	1.48±0.121^b^	0.124±0.005^b^
Cr+EBL	4.87±0.044^a^	2.89±0.061^a^	0.234±0.015^a^
Cr+Spd	3.98±0.034^a^	2.83±0.312^a^	0.189±0.010^a^
Cr+Spd+EBL	4.78±0.101^a^	3.27±0.099^a^	0.263±0.020^a^

Data are presented as mean ± SE. Different superscripted letters (a, b) within a column indicate significant difference from each other in all combinations (Tukey's test, p<0.05).

### EBL and Spd influence endogenous profiles of PGRs under Cr-stress

#### EBL and Spd modulate PA profile under Cr-stress

PA profile of a plant tissue is an indicator of biological or non-biological stress imposed on it. In the next line of our study, we examined the effects of EBL and/or Spd treatments on the endogenous levels of Put and Spd, two representative PAs, in the Cr-stressed seedlings. Additionally, we also examined the expression of *RsADC* and *RsSPDS* genes involved in biosynthesis of Put and Spd, respectively, as a mean to explain the changes in Put and Spd profiles in Cr-stressed plants treated with EBL and/or Spd.

**Figure 1:**
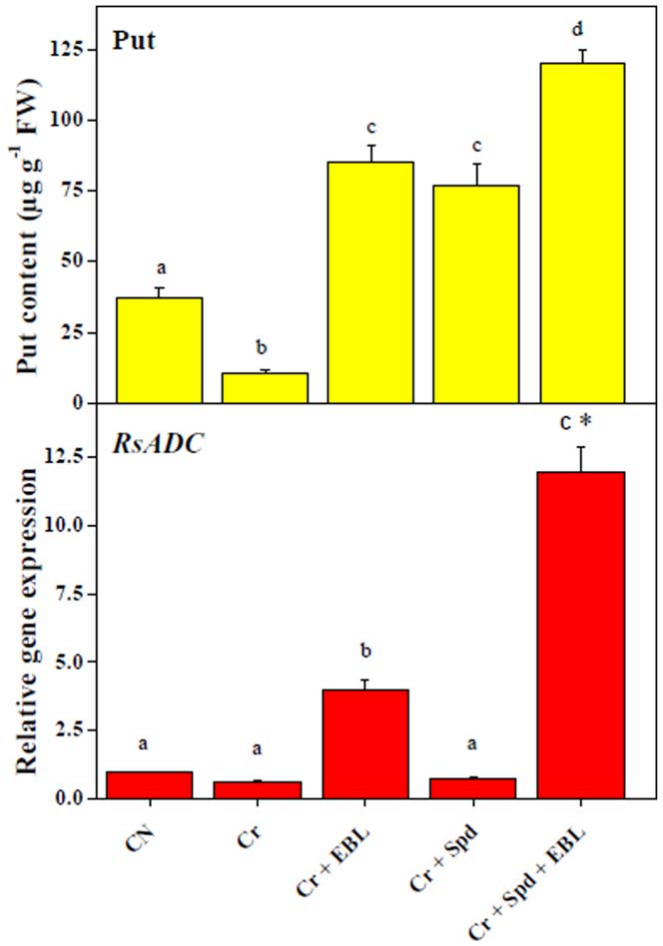
Endogenous levels of Put (µg g^−1^ FW) and relative expression of *RsADC* in 7-d-old radish seedlings under Cr-stress without or with EBL and/or Spd. Data are presented as mean ± SE. Different superscripted letters (a, b, c, d) indicate significant difference from each other in all combinations (Tukey's test, p<0.05). Asterisk “*” indicates significant difference for EBL+Spd vs. EBL under Cr-stress (Tukey's test, p<0.05).

With regard of the endogenous Put content, about 2.36-fold reduction in Put level was observed under Cr-stress over untreated control. The downfall in Put titer in Cr-stressed seedlings might be associated with 2-fold downregulation of *RsADC* expression when compared with untreated control (Figure 1). Elevated Put level (8.02-fold) for EBL treatment alone under Cr-stress might be linked to a 6.3-fold increase in *RsADC* expression in comparison with Cr-treatment only. However, it was interesting to observe that 7.2-fold increase in Put titer for Spd alone under Cr-stress was associated with only 1.17-fold increase in *RsADC* expression over Cr-stress alone. This irregular behavior in *RsADC* expression may be attributed to unknown gene family structure in radish. Co-application of EBL and Spd was found to enhance Put concentration by 11.38-fold, perhaps through significant upregulation of *RsADC* (18.8-fold) expression, in comparison with Cr-treatment alone (Figure 1). Our data indicate that EBL and Spd co-application has higher impact on Put biosynthesis than their independent applications, and the increase in Put levels is, at least in part, associated with the upregulation of the *RsADC* gene.

**Figure 2:**
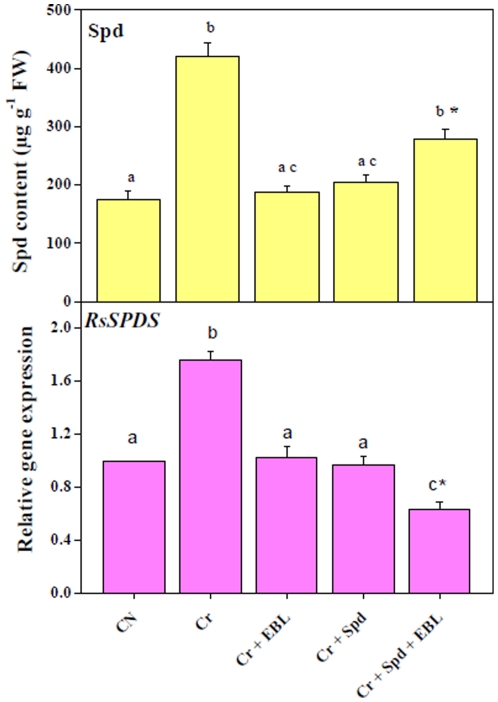
Endogenous levels of Spd (µg g^−1^ FW) and relative expression of *RsSPDS* in 7-d-old radish seedlings under Cr-stress without or with EBL and/or Spd. Data are presented as mean ± SE. Different superscripted letters (a, b, c) indicate significant difference from each other in all combinations (Tukey's test, p<0.05). Asterisk “*” indicates significant difference for EBL+Spd vs. EBL under Cr-stress (Tukey's test, p<0.05).

As for the Spd content, we recorded about 2.39-fold increase in Spd content in Cr-stressed seedlings, which might be attributed to 1.76-fold upregulation of *RsSPDS* expression when compared with untreated control (Figure 2). Application of EBL and Spd resulted in a reduction in Spd levels by 2.25-fold and 2.08-fold, respectively, in Cr-treated seedlings. On the other hand, co-application of EBL and Spd showed only a 1.51-fold decline in Spd concentration over Cr-stressed sample, suggesting that co-application of EBL and Spd does not have better effect on reduction of Spd profile in Cr-stressed seedlings than individual applications of either EBL or Spd (Figure 2, upper panel). The decreases in the Spd contents under the treatments of Cr-stressed seedlings with EBL and/or Spd might be linked, at least in part, to the downregulation of the *RsSPDS* gene (Figure 2, lower panel).

#### EBL and Spd improve endogenous IAA and ABA under Cr-stress

**Figure 3:**
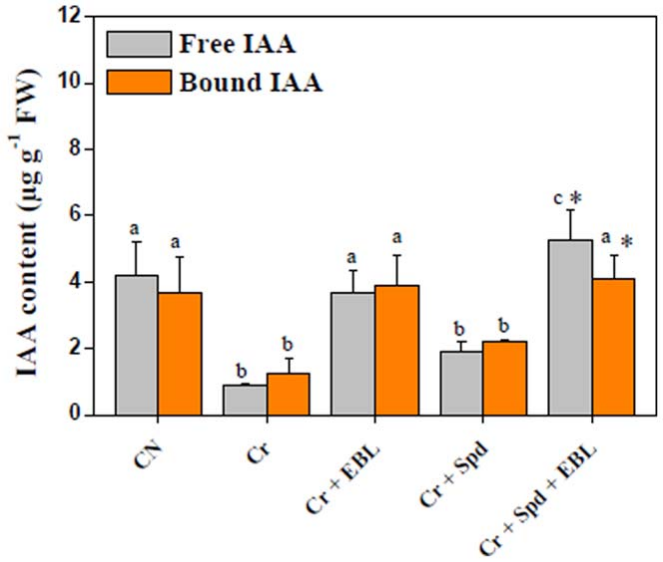
Endogenous levels of free and bound IAA (µg g^−1^ FW) in 7-d-old radish seedlings under Cr-stress without or with EBL and/or Spd. Data are presented as mean ± SE. Different superscripted letters (a, b, c) indicate significant difference from each other in all combinations (Tukey's test, p<0.05). Asterisk “*”indicate significant difference for EBL+Spd vs. EBL under Cr-stress (Tukey's test, p<0.05).

Cr-stressed seedlings showed significant reduction in both free (4.71-fold) and bound IAA (2.98-fold) contents over untreated control (Figure 3). EBL and Cr-stress treatment was able to improve free (4.12-fold) and bound (3.14-fold) IAA levels when compared with Cr-stress treatment alone. Individual application of Spd with Cr-solution brought 2.12-fold and 1.78-fold enhancement in titers of free and bound IAA, respectively, over Cr-stressed seedlings (Figure 3). Further enhancement in both free (5.89-fold) and bound IAA (3.32-fold) concentrations were also recorded for Cr-solution enriched with EBL and Spd over Cr- treatment alone. Our results indicate that EBL and Spd co-application can improve the endogenous IAA levels more significantly than their independent treatments under Cr-stress.

**Figure 4:**
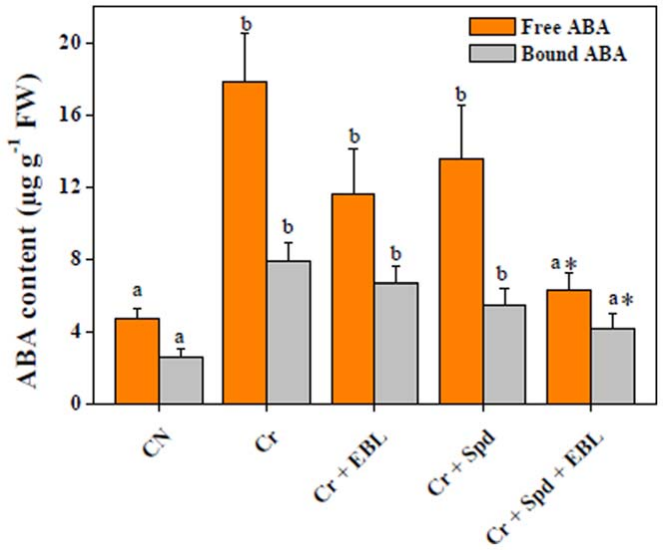
Endogenous levels of free and bound ABA (µg g^−1^ FW) in 7-d-old radish seedlings under Cr-stress without or with EBL and/or Spd. Data are presented as mean ± SE. Different superscripted letters (a, b) indicate significant difference from each other in all combinations (Tukey's test, p<0.05). Asterisk “*”indicates significant difference for EBL+Spd vs. EBL under Cr-stress (Tukey's test, p<0.05).

Seedlings subjected to Cr-stress showed a significant increase in concentrations of free (3.81-fold) and bound (3.08-fold) ABA when compared with untreated control (Figure 4). No significant reduction in free and bound ABA levels was observed for Cr-solution enriched with either EBL or Spd in comparison with Cr-stress alone. However, co-application of EBL and Spd with Cr-solution was able to lower both free (2.81-fold) and bound (1.91-fold) ABA levels when compared with Cr treatment only (Figure 4). Our data suggest that co-application of EBL and Spd has more pronounced effect on ABA titers than their individual applications under Cr-stress.

### EBL and Spd modulate antioxidant parameters to alleviate Cr-stress

#### EBL and Spd improve antioxidant profiles under Cr-stress

Antioxidants play important roles in oxidative stress management in plants. Their titers determine the dietetic values of a food item and are directly correlated with the ability of a plant to cope with abiotic stress to which it is exposed. Evaluation of the parameters below will allow us to examine the impact of EBL and Spd applications, individually or together, on the antioxidant volumes of radish seedlings under Cr-stress.

About 1.68-fold increase in GSH content was noted in Cr-stressed seedlings over untreated control. Seedlings fed with only EBL plus Cr-solution showed an increase in GSH level by1.95-fold when compared with Cr-stressed seedlings (Table 2). Addition of Spd to Cr-solution was also noted to improve GSH level by 1.87-fold over Cr-stress alone. Simultaneous application of EBL and Spd with Cr-solution was able to induce GSH level by 3.15-fold in comparison with Cr-treatment alone, indicating that co-application of EBL and Spd has a more significant effect than their individual applications on the improvement of GSH content in Cr-stressed seedlings.

**Table 2:** Effect of EBL and/or Spd on endogenous profiles of antioxidants in radish seedlings under Cr-stress.

Treatment	GSH(µmol g^−1^ FW)	ASA(µg g^−1^ FW)	PL(µmol g^−1^ FW)	GB(µmol g^−1^ FW)	TP(µg g^−1^ FW)
Control	13.14±1.68^a^	0.67±0.14^a^	1.29±0.095^a^	15.88±1.13^a^	33.15±1.13^a^
Cr	22.16±2.63^b^	2.15±0.39^b^	3.67±0.096^b^	38.16±3.58^b^	12.21±0.84^b^
Cr+EBL	43.18±1.85^c^	3.19±0.23^b^	4.62±0.096^b^	59.18±1.41^c^	23.19±2.98^a^
Cr+Spd	41.56±3.59^c^	3.41±0.35^b^	3.87±0.231^a^	46.15±1.68^b^	25.71±1.14^a^
Cr+Spd+EBL	69.88±3.45^d§^	4.77±0.34^c*^	5.21±0.098^c§^	69.58±2.36^d§^	29.45±1.31^a^

Data are presented as mean ± SE. Different superscripted letters (a, b, c, d) within a column indicate significant difference from each other in all combinations (Tukey's test, p<0.05). Symbols “*” and “^§^” indicate significant difference in a given column for EBL+Spd vs. EBL and Spd+EBL vs. Spd under Cr-stress, respectively (Tukey's test, p<0.05). GSH, glutathione; ASA, ascorbic acid; PL, proline; GB, glycine betaine; TP, total phenols.

Cr-stress was able to promote the titer of ASA significantly (3.20-fold) over untreated control. Application of only EBL to Cr-stressed seedlings showed rise in ASA level by 1.48-fold in comparison with Cr-stressed seedlings alone (Table 2). Supplementation of Spd to Cr- solution was found to enhance ASA content by 1.58-fold over Cr-stressed seedlings. Seedlings treated with Cr-solution supplemented with EBL plus Spd were observed to induce ASA level by 2.21-fold when compared with seedlings treated with Cr only, suggesting the better effect of EBL and Spd co-application than their individual applications in induction of ASA levels in radish seedlings under Cr-stress.

About 2.84-fold increase in PL content under Cr-stress seedlings was noted in comparison with untreated control (Table 2). No significant increase in PL content was noted for EBL or Spd added individually to Cr-solution under Cr-stress when compared with Cr-stress alone. However, seedlings treated with EBL and Spd plus Cr-solution were able to increase the PL level by 1.42-fold over Cr-stressed seedlings.

A significant increase in GB content by 2.40-fold was noted in Cr-stressed seedlings when compared with untreated control (Table 2). Seedlings treated with EBL plus Cr-solution showed 1.55-fold increase in GB level over Cr-treatment alone. Individual Spd application with Cr-solution was observed to improve GB content by 1.21-fold when compared with addition of Cr-solution alone. Combination of EBL and Spd when given with Cr-solution was found to increase GB level by 1.82-fold, more effectively than EBL or Spd given alone to Cr-stressed seedlings, in treated radish plants.

Overconsumption of TP in scavenging ROS induced by Cr-stress was reflected with lower values of TP content. Therefore, 2.71-fold decrease in TP level was observed in Cr-stressed seedlings over untreated control seedlings (Table 2). Cr-solution enriched with only EBL showed 1.9-fold increase in TP content when compared with Cr-stress alone. Treatment of Spd alone with Cr-stress improved TP level by 2.10-fold over Cr-stressed seedlings. However, co-application of EBL and Spd with Cr-solution was able to enhance TP content by 2.41-fold in comparison with only Cr-treatment.

Taken together, our data indicate that co-application of EBL and Spd can more effectively improve the levels of antioxidants than their individual applications in Cr-stressed seedlings.

#### EBL and Spd alter activities of antioxidant enzymes to boost mitigation of Cr-stress

About 2.2-fold decrease in whole protein content was noted in Cr-stressed seedlings when compared with untreated control (Table 3). On the other hand, treatment with EBL but not Spd significantly increased the protein content in Cr-stressed seedlings (∼75% of that of the untreated control), suggesting that EBL can improve protein synthesis under Cr-stress. Co-application of EBL and Spd did not show better enhancing effect on the protein synthesis in Cr-stressed seedlings in comparison with EBL supplementation alone, confirming that EBL but not Spd can alleviate the negative effect of Cr-stress on protein synthesis in radish seedlings (Table 3).

**Table 3:** Effect of EBL and/or Spd on protein content and specific activities of antioxidant enzymes in radish seedlings under Cr-stress.

Treatment	Protein(mg g^−1^ FW)	GPOX(µmol min^−1^ mg^−1^ protein)	SOD(mol U mg^−1^protein)	CAT(µmol min^−1^ mg^−1^ protein)	GR(µmol min^−1^ mg^−1^ protein)
Control	73.06±3.02^a^	0.26±0.088^a^	0.87±0.135^a^	0.086±0.038^a^	0.76±0.199^a^
Cr	33.13±1.04^b^	1.19±0.065^b^	5.74±0.145^b^	0.753±0.055^b^	6.15±0.189^b^
Cr+EBL	54.58±2.25^a^	0.91±0.054^b^	6.56±0.263^b^	0.216±0.053^c^	1.19±0.149^c^
Cr+Spd	43.56±2.17^b^	0.39±0.037^a^	6.26±0.244^b^	0.106±0.031^a^	7.12±0.255^b^
Cr+Spd+EBL	53.61±1.28^b^	0.78±0.052^b§^	8.92±0.015^c*§^	0.375±0.005^c§^	9.16±0.117^d*^

Data are presented as mean ± SE. Different superscripted letters (a, b, c, d) within a column indicate significant difference from each other in all combinations (Tukey's test, p<0.05). Symbols “*” and “^§^” indicate significant difference in a given column for EBL+Spd vs. EBL and Spd+EBL vs. Spd under Cr-stress, respectively (Tukey's test, p<0.05). GPOX, guaiacol peroxidase; SOD, superoxide dismutase; CAT, catalase; GR, glutathione reductase.

We recorded a significant increase in GPOX activity (4.57-fold) in Cr-stressed seedlings in comparison with untreated control. A small decrease in GPOX activity (1.30-fold) for EBL treatment was observed under Cr-stress when compared with Cr-stress alone. Enrichment of Cr-solution with only Spd was found to lower GPOX activity by 3.05-fold over Cr-treatment alone (Table 3). Supplementation of EBL and Spd to Cr-solution was able to reduce GPOX activity by only 1.52-fold compared with Cr-stress alone, suggesting a complex effect of co-application of EBL and Spd on modulation of GPOX activity.

A sharp increase in SOD activity (6.59-fold) over untreated control was noted under Cr-stress. No significant increase in SOD activity was recorded for individual applications of EBL and Spd with Cr-solution when compared with only Cr-treatment (Table 3). However, EBL and Spd applied together enhanced SOD activity (1.55-fold) more efficiently than their individual applications under Cr-stress.

About 8.75-fold increase in CAT activity was recorded in Cr-stressed seedlings in comparison with untreated control (Table 3). Addition of only EBL as supplement to Cr-solution lowered the CAT activity by a significant figure (2.89-fold) over Cr-stressed seedlings alone. Seedlings treated with Cr-solution supplemented with Spd showed 7.10-fold decrease in CAT activity when compared with Cr-stressed seedlings. Furthermore, supplementation of EBL and Spd to Cr-solution could reduce CAT activity by 2-fold in comparison with Cr-treatment alone.

A significant increase in GR activity (8.03-fold) was recorded in Cr-treated seedlings when compared with untreated control (Table 3). Significant reduction (5.16-fold) in GR activity was observed for Cr-solution supplemented with EBL in comparison with Cr-stressed seedlings. Individual application of Spd with Cr-solution insignificantly influenced GR activity over Cr-stress (Table 3). However, co-application of EBL and Spd plus Cr-solution improved GR activity by 1.49-fold over Cr-treatment alone, suggesting the complex nature of interaction between EBL and Spd in modulation of GR activity.

### EBL and Spd modulate stress indices to improve Cr-stress tolerance

Stress indices reflect oxidative stress levels and the damages caused by stresses. Therefore, the evaluation of stress indicators against EBL and Spd under Cr-stress may be used to learn the impact of BRs and PAs in improvement of Cr-stress tolerance in radish.

#### Lipid peroxidation

Membrane damage caused by Cr-stress was indicated with significant increase in MDA level (5.58-fold) as compared with untreated control (Table 4). Application of only EBL supplemented with Cr-solution was able to reduce MDA content (1.72-fold) over Cr-stress alone. Individual application of Spd under Cr-stress was found to reduce MDA content by 1.86-fold in comparison with Cr-treatment alone. Seedlings co-applied with EBL and Spd enriched to Cr-solution showed significant reduction in MDA content (2.30-fold) when compared with only addition of Cr-solution (Table 4).

**Table 4:** Effect of EBL and/or Spd on endogenous levels of stress indicators in radish seedlings under Cr-stress.

Treatment	MDA(µmol g^−1^ FW)	PC(µmol g^−1^ FW)	TSS(µg g^−1^ FW)	Chl a(µg g^−1^ FW)	Chl b(µg g^−1^ FW)	Cart(µg g^−1^ FW)	H_2_O_2_(µmol g^−1^ FW)
Control	0.89±0.193^a^	23.12±2.35^a^	56.67±2.12^a^	57.21±3.82^a^	51.42±2.06^a^	32.45±2.35^a^	25.39±3.42^a^
Cr	4.97±0.267^b^	48.18±1.74^b^	20.21±0.92^b^	22.13±2.32^b^	19.72±1.52^b^	17.76±6.72^b^	205.14±9.13^b^
Cr+EBL	2.88±0.118^c^	54.19±3.55^b^	28.19±2.09^b^	45.62±1.01^a^	39.98±1.89^a^	43.62±2.12^c^	115.39±6.21^c^
Cr+Spd	2.67±0.153^c^	63.73±4.34^b^	26.23±1.09^b^	34.97±2.22^b^	33.28±1.34^c^	27.86±1.96^a^	129.19±5.49^c^
Cr+Spd+EBL	2.16±0.191^c^	73.62±4.32^c*^	41.17±2.13^c*§^	47.78±2.67^b§^	43.69±2.08^a§^	35.83±2.67^a^	89.02±6.79^c§^

Data are presented as mean ± SE. Different superscripted letters (a, b, c) within a column indicate significant difference from each other in all combinations (Tukey's test, p<0.05). Symbols “*” and “^§^” indicate significant difference in a given column for EBL+Spd vs. EBL and Spd+EBL vs. Spd under Cr-stress, respectively (Tukey's test, p<0.05). MDA, malondialdehyde; PC, phytochelatins; TSS, total soluble sugars; Chl a, chlorophyll a; Chl b, chlorophyll b; Cart, carotenoids.

#### Phytochelatins (PC)

About 2.08-fold increase in PC content was recorded under Cr-stress when compared with untreated control. Meager increase in PC level by 1.12-fold was noted for only EBL supplemented with Cr-solution than Cr-stress alone (Table 4). Individual application of Spd to Cr-stressed seedlings revealed a slight rise (1.32-fold) in PC content over Cr-solution. Co-application of EBL and Spd to Cr-solution was more effective (1.59-fold over Cr- treatment alone) than their individual applications in enhancing PC content in Cr-stressed seedlings (Table 4).

#### Total soluble sugars (TSS)

Seedlings under Cr-stress showed significant impact on TSS. A reduction in TSS by 2.80-fold under Cr-stress was recorded in comparison with untreated control (Table 4). Seedlings fed with EBL plus Cr-solution increased TSS by 1.39-fold over Cr-stressed seedlings. Addition of Spd to Cr-solution was able to improve TSS by 1.29-fold in comparison to Cr-stress alone. However, supplementation of both EBL and Spd to Cr-solution showed a significant increase in TSS (2.16-fold) when compared with Cr-treatment alone.

#### Photosynthetic pigments (PPs)

About 2.58-, 2.60- and 1.82-fold decreases in contents of chlorophyll a (Chl a), chlorophyll b (Chl b) and carotenoids (Cart), respectively, were noted in Cr-stressed seedlings when compared with untreated control (Table 4). Addition of EBL to Cr-solution was able to improve contents of Chl a (2.06-fold), Chl b (2.02-fold) and Cart (2.45-fold) in comparison with Cr-treatment alone. Supplementation of Spd to Cr-solution also raised contents of Chl a (1.58-fold), Chl b (1.68-fold) and Cart (1.56-fold) compared with Cr- treatment alone. It was further noted that co-application of EBL and Spd to Cr-solution could improve contents of Chl a (2.15-fold), Chl b (2.21-fold) and Cart (2.01-fold) compared with Cr-solution alone (Table 4).

#### H_2_O_2_ content

H_2_O_2_ level in a plant tissue indicates the severity of oxidative stress. Present investigation recorded enhanced level of H_2_O_2_ (8.07-fold) in Cr-stressed seedlings when compared with untreated control (Table 4). Seedlings treated with EBL plus Cr-solution had lower H_2_O_2_ level (1.77-fold) than Cr-stressed seedlings. Seedlings fed with Spd plus Cr-solution showed a 1.58-fold decline in H_2_O_2_ level in comparison with Cr-treatment alone. Moreover, enrichment of Cr-solution with EBL plus Spd was also noted to reduce H_2_O_2_ content (2.30-fold) when compared with Cr-solution alone (Table 4).

These data suggest that treatments with EBL and Spd, either together or alone, can reduce oxidative stress level in radish plants subjected to Cr-stress, and co-application of EBL and Spd has higher alleviating effect than their individual application.

### Localization of O_2_^−^ and H_2_O_2_

**Figure 5:**
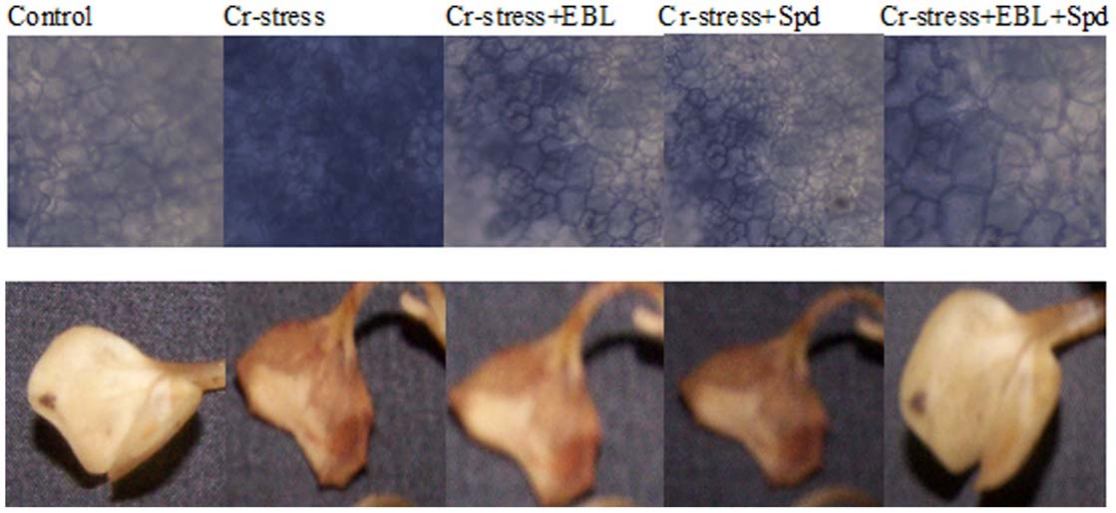
Localization of O_2_^−^ with NBT staining (upper panel) and H_2_O_2_ with DAB staining (lower panel) in cotyledonary leaves of 7-d-old radish seedlings under Cr-stress without or with EBL and /or Spd.

The NBT and DAB staining procedures showed significant production of O_2_^−^ (Figure 5, upper panel) and H_2_O_2_ (Figure 5, lower panel), respectively, in cotyledonary leaves of radish seedlings, under Cr-stress in comparison with untreated control. However, both the staining procedures showed that applications of EBL and Spd, either together or alone, lowered the production of O_2_^−^ and H_2_O_2_ in Cr-stressed cotyledonary leaves, and the co-aplication of EBL and Spd has higher scavenging effect than their individual applications (Figure 5).

### Effect of EBL and Spd applications on NADPH oxidase activity

**Figure 6:**
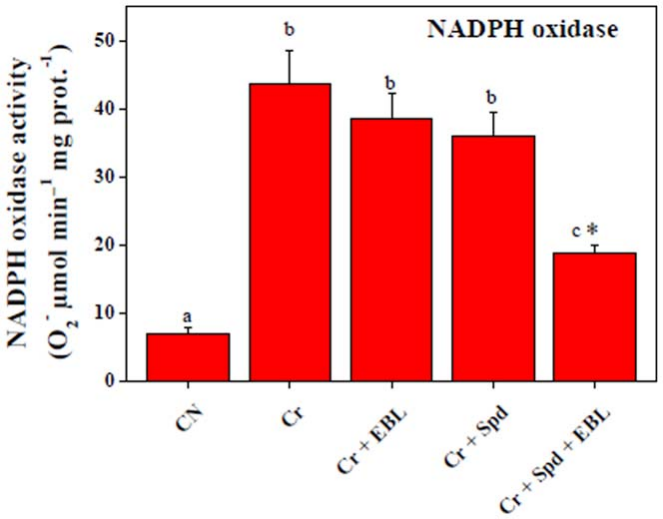
NADPH oxidase activity (O_2_^−2^ µmol min^−1^ mg prot.^−1^) in 7-d-old radish seedlings under Cr-stress without or with EBL and /or Spd. Data are presented as mean ± SE. Different superscripted letters (a, b, c) indicate significant difference from each other in all combinations (Tukey's test, p<0.05). Asterisk “*”indicates significant difference for EBL+Spd vs. EBL under Cr-stress (Tukey's test, p<0.05).

Activity of NADPH oxidase is directly related to the production of H_2_O_2_ in the plasma membrane. About 6.24-fold increase in NADPH oxidase activity was observed in Cr-stressed seedlings in comparison with untreated control (Figure 6). No significant change in NADPH oxidase activity was noted for seedlings applied with EBL and Spd separately under Cr-stress. However, significant reduction in NADPH oxidase activity (2.32-fold) was observed for co-application of EBL and Spd with Cr-solution in comparison with Cr-treatment alone. The results together indicate that EBL and Spd co-application is more effective in modulating NADPH oxidase activity than their independent applications (Figure 6).

### EBL and Spd reduce ion leakage under Cr-stress

**Figure 7:**
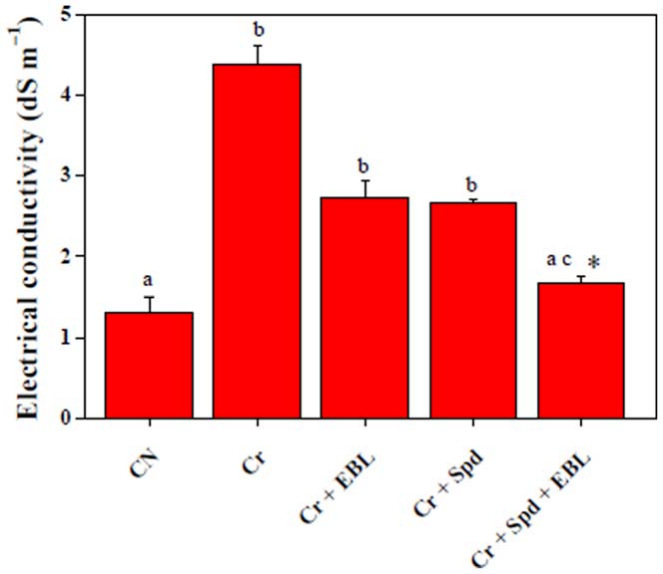
Ion leakage (dS m^−1^) of 7-d-old radish seedlings under Cr-stress without or with EBL and /or Spd. Data are presented as mean ± SE. Different superscripted letters (a, b, c) indicate significant difference from each other in all combinations (Tukey's test, p<0.05). Asterisk “*”indicates significant difference for EBL+Spd vs. EBL under Cr-stress (Tukey's test, p<0.05).

Membrane permeability is severely affected by ROS, which was also observed under Cr-stress (Figure 7). About 3.34-fold increase in electrical conductivity (EC) was noted in Cr-stressed seedlings over untreated control. Application of EBL to Cr-stressed seedlings showed significant decline in EC value (1.6-fold) when compared with Cr-treatment alone. Cr-stressed seedlings fed with Spd also exhibited significant decrease in EC value (1.64-fold) in comparison with Cr-stressed sample. Supplementation of EBL and Spd together to Cr-stressed seedlings showed higher decline (2.62-fold over Cr-treated sample) in EC value than their individual applications.

### Impacts of EBL and Spd applications on photosystem II (PSII) quantum yield

Significant reduction in quantum yield (Fv/Fm) was recorded in cotyledonary leaves of Cr-stressed seedlings in comparison with untreated control (Table 5). Applications of EBL and Spd, either together or alone, could significantly increase quantum yield in Cr-stressed seedlings. We also observed that co-application of EBL and Spd has better impact on PSII quantum yield than their individual applications under Cr-stress.

**Table 5:** Effects of EBL and Spd on PS II quantum yield (Fv/Fm), Cr-uptake and radical scavenging activities in radish seedlings under Cr-stress.

Treatment	PS IIquantum yield(Fv/Fm)	Cr content(µg g^−1^ DW)	% inhibition of DPPH activity	% inhibition of deoxyribose degradation	% FRAP activity
Control	0.29±0.033^a^	0.08±0.04^a^	35.21±3.82^a^	39.17±4.06^a^	45.15±2.32^a^
Cr	0.11±0.052^b^	1.25±0.09^b^	11.31±1.18^b^	13.12±3.55^b^	10.18±1.60^b^
Cr+EBL	0.18±0.052^b^	0.12±0.01^a^	32.14±3.14^a^	22.45±6.03^a^	31.23±3.21^a^
Cr+Spd	0.13±0.038^b^	0.25±0.01^c^	29.14±7.69^a^	34.12±5.85^a^	30.23±3.54^a^
Cr+Spd+EBL	0.21±0.032^a§^	0.15±0.01^a§^	33.16±5.58^a^	38.13±6.35^a*^	42.13±4.10^c*§^

Data are presented as mean ± SE. Different superscripted letters (a, b, c) within a column indicate significant difference from each other in all combinations (Tukey's test, p<0.05). Symbols “*” and “^§^” indicate significant difference in a given column for EBL+Spd vs. EBL and Spd+EBL vs. Spd under Cr-stress, respectively (Tukey's test, p<0.05). PS II, photosystem II; DPPH, 1,1-diphenylpicrylhydrazyl; FRAP, Fe^2+^ ions reducing antioxidant power.

### EBL and Spd co-applications reduce Cr (VI) uptake

Significant uptake of Cr (VI) ions was noted in seedlings subjected to Cr-stress over untreated control (Table 5). We recorded 15.62-fold increase in Cr-content in stressed seedlings when compared with untreated control. Seedlings applied with only EBL enriched to Cr-solution possessed lowered Cr content (10.41-fold) than Cr-stress alone. Individual application of Spd to Cr-treated seedlings had 5-fold reduction in Cr content in comparison with Cr-treatment alone. Supplementation of EBL and Spd to Cr-solution was also observed to lower Cr content (8.33-fold) (Table 5). Our results indicate that co-application of EBL and Spd showed no further improvement in reducing Cr accumulation in Cr-stressed seedlings.

### Effect of EBL and Spd applications on free radical scavenging activity

The screening results of various assays performed to evaluate the effect of EBL and Spd applications, either together or alone, on the free radical scavenging activity are as follows:

#### DPPH assay

Cr-stressed seedlings showed significant downfall (3.11-fold) in their potential to scavenge DPPH radical over untreated control (Table 5). Seedlings fed with Cr-solution enriched with EBL solution showed significant improvement (2.84-fold) in DPPH radical scavenging when compared with Cr-stress alone. About 2.57-fold improvement in radical scavenging was also recorded for seedlings treated with Spd plus Cr-solution when compared with Cr-treatment alone. Application of Cr-solution supplemented with EBL and Spd also showed increase (2.93-fold) in DPPH radical scavenging capacity over Cr-solution alone (Table 5).

#### Deoxyribose assay

Seedlings under Cr-stress showed significant reduction in potential to inhibit deoxyribose degradation (2.98-fold) when compared with untreated control (Table 5). EBL applied with Cr-solution was able to improve inhibitory power of deoxyribose degradation by 2.22-fold over Cr-treatment alone. Application of Spd under Cr-stress also caused an increase in (2.60-fold) inhibitory power over Cr-treatment alone. Combination of EBL plus Spd with Cr-solution could significantly enhance inhibitory potential (2.90-fold) in comparison with that of Cr-stressed seedlings (Table 5).

#### FRAP assay

A significant decrease (4.43-fold) in reducing power was recorded for Cr-treated seedlings when compared with untreated control (Table 5). Application of EBL to Cr solution was found to improve reducing power by 3.06-fold when compared with Cr-solution alone. Reducing power of seedlings treated with both Spd and Cr-solution was improved 2.96-fold over seedlings under Cr-stress only. Moreover, co-application of EBL and Spd enhanced most significantly the reducing power (4.13-fold over Cr-treatment), almost to the untreated control level, in Cr-stressed seedlings.

## Discussion

Last few decades have witnessed emergence of Cr (III or VI) as major environmental pollutant. Among natural forms of Cr, Cr (VI) is highly phytotoxic owing to its greater mobility in soil solution surrounding rhizosphere [1]. All biological systems have devised unique strategies to combat oxidative stress, an outcome of excessive ROS. Active participations of antioxidant system, PC, MT and several osmolytes in metal detoxification have been widely established [7]. BRs have become a popular tool for oxidative stress amelioration [54]. PA involvement in abiotic stress management, particularly metal excess, has given them a status of favorite metal detoxifiers in plants [22]. Though significant amounts of information are available on the individual applications of BRs and PAs in heavy metal stress mitigation, their co-application potential has not been tested for their efficacy to induce metal stress tolerance. Thus, the present investigation is aimed to validate our hypothesis that BRs and PAs may interact to influence metal detoxification system and oxidative stress alleviation in general.

Reduced seedling growth (RL, SL and FW) under Cr (VI) stress might be associated with ability of Cr (VI) to compete for uptake transporters mainly used by indispensable divalent ions (Fe, P and S) which are required for normal plant growth [1]. Combination of EBL and Spd was most effective in enhancing seedling growth parameters under Cr-stress. Improved morphological parameters could be attributed to BR (EBL) ability to regulate cell elongation and divisional activities through the up-regulation of xyloglucan endo-transglycosylase [55]. Improved seedling growth may also be connected with potential of Spd to influence cell elongation process [12]. Recently, a study has indicated the crucial involvement of Spd as a morphogenic determinant of cell fate in male gametophyte of water fern [56]. It is interesting to observe that co-application of EBL and Spd has more pronounced effects on the improvement of seedling growth under Cr-stress, perhaps due to their synergistic or additive effects.

Participations of PGRs in heavy metal detoxification have been widely established [6], [11], [12], [27]. To further test the objective of our hypothesis that EBL and Spd may influence profiles of PGRs (PAs, auxins and ABA) to alleviate metal stress, we determined the endogenous titers of PGRs in Cr-stressed seedlings treated with EBL and/or Spd. PA biosynthesis and catabolism have been directly associated with abiotic stresses [12]. Our results have validated the hypothesis on the influence of EBL and Spd interactions on PA pools and their involvement in Cr-stress mitigation. Enhanced Put content in Cr-stressed seedlings supplemented with EBL might contribute to, at least in part, improvement of seedling growth (Figure 1). In addition, further improvement in Put titers for co-application of EBL and Spd under Cr-stress than their independent treatments suggests an additive or synergistic effect on PA metabolism.

It is well established that the levels of PAs, such as Put and Spd, in a plant tissue increase under various abiotic stresses [20], [57], [58]. Being antioxidant in nature, the increased volume of Spd ascertains ROS and metal detoxification. Therefore, the exogenous application of Spd in our present experimental set up acted as a supplement to further enhance the overall level of Spd to mitigate Cr-induced oxidative stress. Spd is mostly expressed and synthesized in higher quantities under stress and is restored to normal level upon alleviation of stress. In the present study, significant increase in Spd content in Cr-stressed seedlings may be associated with the effort to combat or neutralize ROS generated under Cr-stress through utilization of more basic nature of Spd. We observed that EBL application alone could reduce the Spd titer significantly under Cr-stress. This downfall in Spd by EBL alone was more than the decline in Spd level brought by individual application of Spd under Cr-stress. Moreover, EBL and Spd co-application with Cr-stress could also lower Spd titers, though insignificantly, thus indirectly reflecting the diminution of Cr-stress (Figure 2). Our previous studies also indicated significant impacts of EBL on the endogenous titers of PAs under Cu^2+^ stress [27]. From the above observations, it may be suggested that EBL and Spd co-applications may have additive and/or synergistic interactions to influence endogenous Put and Spd pools through selective modulation of *RsADC* and *RsSPDS* gene expression to minimize the Cr-induced oxidative stress damages in radish.

Enhanced titers of free and bound IAA were recorded in seedlings fed with Cr-solution enriched with EBL alone. No significant increase in either free or bound IAA was observed for Spd application alone under Cr-stress. It was further noticed that co-application of EBL and Spd was more effective in improving the endogenous titers of free and bound IAA than their independent application, resulting in improved seedling growth even under Cr-stress (Figure 3). This may be one of the strategies which EBL uses to enhance metal stress tolerance through increasing the endogenous levels of free and bound IAA. Moreover, active participation of auxins in metal stress alleviation can be supported by the observations of Dimpka et al. [59]. These authors have found stress protective role of auxins with siderophores in heavy metal stress diminution in *Streptomyces spp*.

ABA is a stress hormone protecting plants under abiotic stresses. Homeostasis of ABA endogenous titers is very crucial for the normal metabolic activities, such as opening and closing of stomata, dormancy and senescence. Excess of ABA synthesis leads to closure of stomata with net outcome of reduced photosynthesis [24], [57], [60], [61]. In the present investigation, Cr-stress was found to enhance both free and bound ABA concentrations over untreated control (Figure 4). In our present study augmented ABA profiles under Cr-stress might be connected with reduced seedling growth, associated with enhanced dormancy and low quantum yield recorded in young radish seedlings. Slight decrease in free and bound ABA levels in Cr-stressed seedlings supplemented with either EBL or Spd might partly contribute to improved seedling growth under metal stress tolerance. We observed that co-application of EBL and Spd was able to reduce ABA titers from stressful regime to nearly normal levels (Figure 4), which may be correlated with normalization of metabolic activities by lowering ABA levels. Recently, interactions of BRs and ABA have been shown to improve thermotolerance of *A. thaliana* [11]. Additionally, another independent study has indicated the possible interactions of ABA with PAs to orchestrate PA metabolism and stress responses in grapevine [62].

Improved antioxidant system is a key player in alleviation of oxidative stress posed by ROS [7]. We also verified our hypothesis by checking the effects of EBL and Spd co-applications on the major components of antioxidant system. Modulated concentrations/activities of antioxidants/antioxidant enzymes, such as GSH, ASA, PL, GB and TP, and enzyme activities (GPOX, CAT, SOD and GR), noted in Cr-stressed seedlings demonstrated their active participation in oxidative stress management (Table 2, 3). Application of either EBL or Spd with Cr-stress increased antioxidant titers, with ultimate aim to improve seedling growth under Cr-stress. Furthermore, improvement in antioxidant system in seedlings fed with both EBL and Spd together under Cr-stress was more evident than their individual application. Our findings are in concordance with the observations reported previously [6], which showed improvement/modulation in antioxidant (ASA, GSH and PL) titers and enzyme activities in *R. sativus* upon 28-homobrassinolide treatment under Cr-stress.

Cr-stressed seedlings showed significant downfall in the levels of examined stress indicators, including MDA, TSS, Chl a, Chl b and Cart, and H_2_O_2_, when compared with untreated control (Table 4). Application of EBL and Spd together was able to increase the levels of TSS, Chl a, Chl b, Cart and TP more significantly than their individual treatments, thereby more effectively improving the Cr-stress management capacity of radish seedlings. The active implication of these stress indices as oxidative stress markers has been widely documented in plants [7].

PCs minimize metal toxicity through complex formation and conjugation. Enhanced titers of PCs observed under Cr-stress (Table 4, [10]) were found to be improved in Cr-stressed seedlings fed with either EBL or Spd. Moreover, co-application of EBL and Spd has more prominent effects on PC biosynthesis than their individual applications (Table 4). Furthermore, applications of EBL and Spd, either together or alone, were also able to reduce the Cr (VI) uptake (Table 5), through a less known phenomenon affecting adventitious roots of seedlings [1], [6].

Additionally, radical scavenging assays performed in this study demonstrate strong impact of EBL and Spd applications on the abilities of radish seedlings to scavenge ROS. Reduced values of DPPH, deoxyribose and reducing power assays and higher contents of H_2_O_2_ content were recorded under Cr-stress (Table 5). Positive impact of EBL and Spd alone/together on reduction in ROS volume also revealed Cr-stress alleviation. Both NBT and DAB staining methods also showed that EBL and Spd applications, alone or together, could reduce the production of O_2_^−^ and H_2_O_2_ under Cr-stress (Figure 5). However, applications of EBL and Spd were able to remarkably improve the DPPH, deoxyribose and FRAP values, while significantly reduce H_2_O_2_ content in Cr-stressed seedlings. Co-application of EBL and Spd was found to have better effect on ROS scavenging than their individual applications (Table 5). In addition, EBL and Spd induced decline in H_2_O_2_ content under Cr-stress was associated with a significant decrease in NADPH oxidase activity (Figure 6).

Cr-induced ROS caused severe damages to membrane permeability as shown by increased EC value (Figure 7). However, applications of EBL and Spd, either individually or together, were able to reduce the EC values, perhaps by enhancement of antioxidant system and various stress-related parameters as discussed above. This result indicates the stabilizing effects of EBL and Spd, especially when co-applied, on the membrane permeability under Cr-stress.

### Conclusions

Our results demonstrate that co-application of BRs and PAs is more effective in alleviation of Cr-stress than their individual treatments. Improved Cr-stress mitigation with EBL and Spd co-application involves physiological and molecular interactions in a synergistic and/or additive manner. Therefore, these findings provide a unique and eco-friendly strategy employing interplay of BRs and PAs to overcome heavy metal stress mitigation, and abiotic stress in general, in radish.
